# The Use of Virtual Characters to Assess and Train Non-Verbal Communication in High-Functioning Autism

**DOI:** 10.3389/fnhum.2014.00807

**Published:** 2014-10-15

**Authors:** Alexandra Livia Georgescu, Bojana Kuzmanovic, Daniel Roth, Gary Bente, Kai Vogeley

**Affiliations:** ^1^Department of Psychiatry and Psychotherapy, University Hospital Cologne, Cologne, Germany; ^2^Ethics in the Neurosciences (INM-8), Institute of Neuroscience and Medicine, Research Center Juelich, Juelich, Germany; ^3^Department of Psychology, University of Cologne, Cologne, Germany; ^4^Cognitive Neuroscience (INM-3), Institute of Neuroscience and Medicine, Research Center Juelich, Juelich, Germany

**Keywords:** high-functioning autism, non-verbal behavior, social interaction, virtual reality, virtual characters, social gaze

## Abstract

High-functioning autism (HFA) is a neurodevelopmental disorder, which is characterized by life-long socio-communicative impairments on the one hand and preserved verbal and general learning and memory abilities on the other. One of the areas where particular difficulties are observable is the understanding of non-verbal communication cues. Thus, investigating the underlying psychological processes and neural mechanisms of non-verbal communication in HFA allows a better understanding of this disorder, and potentially enables the development of more efficient forms of psychotherapy and trainings. However, the research on non-verbal information processing in HFA faces several methodological challenges. The use of virtual characters (VCs) helps to overcome such challenges by enabling an ecologically valid experience of social presence, and by providing an experimental platform that can be systematically and fully controlled. To make this field of research accessible to a broader audience, we elaborate in the first part of the review the validity of using VCs in non-verbal behavior research on HFA, and we review current relevant paradigms and findings from social-cognitive neuroscience. In the second part, we argue for the use of VCs as either agents or avatars in the context of “transformed social interactions.” This allows for the implementation of real-time social interaction in virtual experimental settings, which represents a more sensitive measure of socio-communicative impairments in HFA. Finally, we argue that VCs and environments are a valuable assistive, educational and therapeutic tool for HFA.

## Non-Verbal Communication and High-Functioning Autism

### Non-verbal behavior and social cognition

Non-verbal communication constitutes an essential aspect of social cognition. Indeed, non-verbal cues are known to influence person perception and construal processes early during social encounters (Willis and Todorov, 2006) and a large proportion of social meaning is substantially informed by non-verbal cues (Argyle, 1988; Burgoon, 1994). Thus, the investigation of the behavioral and neural correlates of non-verbal behavior processing can deliver valuable insights into social cognition and human communication.

Behavioral research has long been investigating the perception and evaluation of facial or bodily cues (i.e., decoding of emotions and intentions). More recently, the field of social and affective neuroscience has also started investigating the underlying neural mechanisms associated with the social processing of facial and bodily non-verbal cues (in terms of mental state attribution, Gallagher and Frith, 2003; De Gelder and Hortensius, 2014), and also those involved in perceiving meaningful intransitive actions (i.e., non-object directed), be they mimed, expressive, or symbolic (e.g., Gallagher and Frith, 2004; Grèzes et al., 2007; Villarreal et al., 2008). Neurally, this processing is traceable to two main networks in the human brain: the action observation network (AON), associated with human movement perception, and the social neural network (SNN), involved in social-cognitive processing (e.g., Van Overwalle and Baetens, 2009).

### High-functioning autism and non-verbal behavior

Autism spectrum disorders (ASD) are characterized by impairments in communication and reciprocal interaction (World Health Organization, 1993). Socio-communicative deficits in high-functioning autism (HFA) manifest themselves in problems with spontaneously producing, interpreting, and responding to non-verbal cues. More specifically, on the perception side, the intrinsic value and salience of non-verbal cues are reduced in individuals with HFA. They do not spontaneously attend to social information, and are thus less able to intuitively interact in social contexts (Klin et al., 2003). When confronted with non-verbal signals, such as eye gaze, facial expressions, or gestures; individuals with HFA have shown atypical detection (Senju et al., 2005, 2008; Dratsch et al., 2013) and interpretation of such cues (Baron-Cohen, 1995; Baron-Cohen et al., 1997; Uljarevic and Hamilton, 2013) and have difficulties in integrating them for the purpose of an adequate impression formation of others (Kuzmanovic et al., 2011). Generally, they seem to be less affected by them when processing a task, as compared with typically developed control persons (Schwartz et al., 2010; Schilbach et al., 2012), and/or they seem to use atypical strategies for social processing (e.g., Kuzmanovic et al., 2014; Walsh et al., 2014). Furthermore, neuroimaging studies have shown that the SNN, which is involved in conscious mental inference and evaluation of social stimuli (Gallagher and Frith, 2003; Frith, 2007; Van Overwalle and Baetens, 2009), shows a diminished response to the processing of non-verbal social information in HFA (Baron-Cohen et al., 1999; Critchley et al., 2000; Piggot et al., 2004; Pelphrey et al., 2005; Ashwin et al., 2007; Pitskel et al., 2011; Redcay et al., 2012; von dem Hagen et al., 2013; Georgescu et al., 2013; Kuzmanovic et al., 2014).

Thus, non-verbal information may influence social perception, as well as affective and inferential processing, all of which have been demonstrated to be impaired in HFA. In addition, non-verbal behavior exhibits specifically high levels of complexity that are closely related to intuitive cognitive and affective processing. For this reason, investigating non-verbal behavior processing in HFA not only helps to understand (1) social cognition and its underlying neural mechanisms but also (2) the specific cognitive style characteristic of HFA. These insights, in turn, are most valuable for improving supportive therapy and training options, which may improve the lives of affected individuals and their families.

Nevertheless, the investigation of non-verbal behavior faces several basic methodological challenges, which will be elaborated in the next section. Virtual characters (VCs) are introduced as a means to overcome such methodological issues and various experimental implementations are discussed. Finally, we will consider how these implementations have been or could be used in the future for research and training with individuals with HFA.

## Virtual Characters as a Tool for Non-Verbal Behavior Research

### Basic methodological problems in non-verbal behavior research

In contrast to verbal communication, non-verbal cues cannot be readily translated into distinct meanings (Krämer, 2008). In fact, non-verbal behavior is characterized by (a) high dimensional complexity and (b) high processual complexity (Krämer, 2008; Vogeley and Bente, 2010). Dimensional complexity relates to the fact that non-verbal signals are highly context dependent and comprise a simultaneous multichannel activity (Poyatos, 1983). The interpretation of a single non-verbal cue depends on which other verbal, non-verbal, and situational cues precede, co-occur with, or follow it (e.g., Grammer, 1990; Chovil, 1991). Moreover, processual complexity implies that meaningful information is conveyed by dynamic aspects of facial expressions and movements of the head or body, and that subtle spatiotemporal characteristics of perceived behavior can affect the way that non-verbal information is processed (e.g., Birdwhistell, 1970; Grammer et al., 1988, 1999; Krumhuber and Kappas, 2005; Krumhuber et al., 2007; Provost et al., 2008). Therefore, Burgoon et al. (1989) (p. 23) argue for the use of dynamic non-verbal stimuli and suggest that “we need to understand non-verbal communication as an ongoing, dynamic process rather than just a static snapshot of cues or final outcomes at one moment of time.”

Some researchers have used dynamic non-verbal stimuli either by using so-called “thin slices” of people’s behavioral streams (i.e., brief excerpts of behavior, less than 5 min in length; Ambady and Rosenthal, 1992), or by instructing confederates or actors to produce or vary particular aspects of their non-verbal behavior. This approach, however, has its disadvantages due to the fact that implicit movement qualities are both produced and perceived automatically and outside awareness, hence making them difficult to capture and/or control experimentally (Choi et al., 2005). Therefore, a large amount of research to date has investigated non-verbal cues using only static photographs or pictures of, for instance, specific gestures or emotional faces and bodies.

In addition, when using neuroimaging techniques to investigate non-verbal behavior processing, a set of unique challenges with respect to ecological validity emerges. David (2012) states that participants are restricted in the movements they can make in order to prevent artifacts in the recording of neural data. Therefore, neuroimaging paradigms are rather limited in terms of how much they allow participants to engage with a social stimulus. Furthermore, increasing ecological validity involves increasing complexity of the stimulus material and/or task demands, which raise the question concerning what a “neural correlate” actually really reflects (David, 2012). Therefore, oftentimes for experiments, social scripts and stimuli have to be reduced and presented repeatedly, in order to increase statistical power, yet then they also lack ecological validity and may lead to habituation and/or expectancy effects.

To sum up, the investigation of the processing of non-verbal behavior meets several basic methodological challenges, some inherent in the nature of the stimulus (i.e., experimental control) and others caused by technical restrictions (i.e., ecological validity).

### Virtual characters offer a good compromise between experimental control and ecological validity

The abovementioned challenges can be overcome by using anthropomorphic VCs. These are artificial characters, which have realistic human features, can be either static or dynamic and can either be animated by using key framing or motion-capturing techniques. Moreover, VCs are a medium through which virtual interaction partners can be expressed. In this line, research distinguishes between two possible virtual representations of human beings in an interacting context, which differ in terms of their level of agency (Bailenson and Blascovich, 2004; Bailenson et al., 2006; von der Pütten et al., 2010b): (1) agents (i.e., a digital model of a person, which is driven by a computer algorithm) and (2) avatars (i.e., a digital model of a person, which is controlled by a real human in real time). VCs have the advantage of realistic behavior capabilities on the one hand, and systematic manipulability on the other, hence allowing the simultaneous increase of both experimental control and ecological validity (Vogeley and Bente, 2010; Bohil et al., 2011). Moreover, they provide the option to control and investigate body motion independently from body shape, a methodological advantage termed as “plasticity” by Bente and Krämer (2011). This possibility of independently masking or transforming aspects of both appearance and behavior is essential in order to disentangle top-down effects of appearance from bottom-up effects of behavior (Bente et al., 2008).

The most important prerequisite for using VCs for non-verbal behavior research is that they are veridical and convincing and that they are able to evoke impressions, attributions, and reactions in an observer that are comparable to those evoked by real human beings (Krämer, 2008; Vogeley and Bente, 2010). Indeed, the validity of VCs in non-verbal behavior research has been amply demonstrated in both behavioral and neuroimaging studies, and there is consistent evidence that VCs are perceived comparably to real human beings. For example, a series of studies have shown that person perception ratings based on the non-verbal behavior of videotaped human beings do not differ significantly from those based on the identical movements performed by VCs (Bente et al., 2001). Moreover, virtual emotional facial and bodily expressions are recognized as accurately as natural ones (Dyck et al., 2008; McDonnell et al., 2008), and recent functional neuroimaging research demonstrated that facial animations of emotional virtual faces also evoke brain responses comparable to those evoked by real human faces, specifically in the amygdala (Moser et al., 2007), a region robustly associated with social processing (Adolphs et al., 1998).

### Methodological implementations and setups for the use of virtual characters

**Figure 1:**
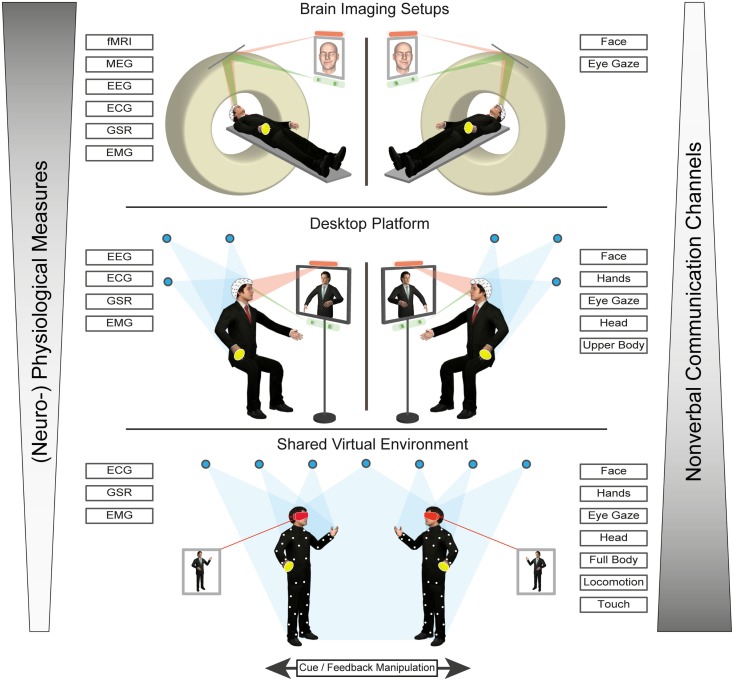
**Illustration of a prototype of a multilayer avatar platform to study production and perception of non-verbal cues in “online” social interaction paradigms**. It shows that increasing ecological realism and communication bandwidth measurement (comprising eyes, face, gaze, movement and even touch) comes at the cost of measurement channels (such as sensors and devices) and that a tradeoff has to be achieved, based on the research question of interest.

Virtual characters can be used within a variety of virtual reality (VR) systems, which can differ in terms of their immersive potential. Immersion refers to the degree of sensory stimulation through the system on the one hand and the sensitivity of the system to motor inputs, on the other (Biocca et al., 2003). Thus, the level of immersion of a VR system is determined by the number of sensory and motor channels connected to the virtual environment (VE) (Biocca et al., 2003). For instance, desktop virtual environments (DVEs) typically involve a user viewing a VE through a computer screen (Bente and Krämer, 2011). While a participant can interact with the environment, using common input devices, such as keyboard, mouse, joystick, or touchscreen, the interaction does not include a high degree of immersion. Some examples for the use of DVEs would be the interaction within online communities like “Second Life” or various desktop-based training programs. Nevertheless, for research investigating not just the perception but also the production side of non-verbal behavior, there is an important consideration to be made: classic DVE setups often require a conscious decision by the user to launch the non-verbal cue via discrete input options, such as clicking a button or hitting a key (Bente and Krämer, 2011). Thus, the sender of a non-verbal message would be more self-aware, as they would have to consciously choose what to display and when to do it. Furthermore, the number of non-verbal signals produced is restricted as cognitive resources of the sender are limited (Bente and Krämer, 2011). Nevertheless, even though they lack peripheral vision, DVEs can increase their immersive potential, by making use of stereoscopic monitors and/or head tracking (e.g., Fish Tank VR, Ware et al., 1993). Another example, would be the virtual communication environment [VCE, by Bente and Krämer (2011), see also the desktop platform illustrated in Figure 1], which is a DVE paradigm that conveys in real time a wide range of non-verbal cues via eye and motion tracking.

The so-called immersive virtual environments (IVEs) typically have a higher immersion potential, compared to classic DVEs, which can be achieved for instance by including continuous real-time tracking of a user’s movements with high degrees of freedom and/or by engaging peripheral in addition to central vision (Bente and Krämer, 2011). Such systems are better at capturing and transmitting a broader range of behavior and allowing for a spontaneous and subconscious usage of non-verbal cues (Bente and Krämer, 2011). IVEs may make use of different display and tracking solutions. For instance, curved screen projections, such as Powerwalls or Tiled Walls (e.g., HEyeWall; Santos et al., 2007) use a combination of multiple projectors or LCD panels to increase the overall display size and resolution and display monoscopic or stereoscopic content. Some systems are equipped with a head mounted display (HMD, a visual display worn as a type of helmet) and are directly tracking movements of the user’s head and/or body to duplicate them within the VE (Bohil et al., 2011). Another type of IVE refers to open display systems, where the user is inside a room or sphere, the surface of which is a seamless display system such as CAVEs (Cruz-Neira et al., 1993) or fulldomes (Bohil et al., 2011). To increase immersiveness, all these setups may use devices to track locomotion (location trackers), hand movements (data gloves or 3D mice), and body motion (motion capture). It has to be noted that, due to end-to-end time lags (between users actions and the correspondent display changes) such immersive technologies can cause “virtual reality induced symptoms and effects” (VRISE, Sharples et al., 2008, previously also referred to as “cyber-sickness,” LaViola, 2000). This issue has to be considered when designing experiments in IVEs.

Whether DVEs or IVEs are used, and which level of immersion is adopted for research purposes, usually depends on the research question, as well as on the budget and accessibility of the technology.

### Recent developments in behavioral paradigms using virtual characters as real-time interaction partners

Most behavioral and neuroimaging studies on non-verbal behavior processing using VCs have used DVEs and observational paradigms and have focused mostly on the perception side of social cognition. In such paradigms, a participant merely observes and evaluates non-verbal cues performed by a virtual other on a screen, without being involved in an interaction with them (e.g., Schilbach et al., 2006; Kuzmanovic et al., 2009, 2012). De Gelder and Hortensius (2014) (p. 160) explain that in observational paradigms, which are also called “offline” paradigms (Pfeiffer et al., 2013a; Schilbach et al., 2013; Schilbach, 2014), the “person observed is not influenced by the way his/her actions are perceived by others. On the other hand, the observer does not get any feedback or insight from his/her correct perception; neither does he/she suffer the consequences of misperception.” In the same line, Patterson (1994) highlights that social interaction consists of both person perception and behavior production simultaneously. Thus, there is growing consent that observational paradigms alone are insufficient for a comprehensive understanding of the neural mechanisms of social cognition, and researchers have recently been arguing for a paradigm shift toward “online,” interactive experimental designs [Hari and Kujala, 2009; Dumas, 2011; Konvalinka and Roepstorff, 2012; Pfeiffer et al., 2013a; Schilbach et al., 2013; Schilbach, 2014; but see also Przyrembel et al. (2012) for a more cautious review highlighting current limitations from a philosophical, psychological, and neuroscientific perspective].

Confirmation for the validity of VCs for “online,” interactive paradigms comes from VR research, where agents have been observed to evoke comparable social effects and behaviors as during the interaction with a real human (e.g., Sproull et al., 1996; Nass and Moon, 2000; Hoyt et al., 2003; Park and Catrambone, 2007). Moreover, VR research has repeatedly confirmed that social interactions in VEs are governed by the same social norms as social interactions in the real world, and that social norms relating to gender, interpersonal distance, approach behavior, and eye gaze can be transferred to VEs (Bailenson et al., 2001; Garau et al., 2005; Yee et al., 2007). However, one of the shortcomings of “online” social interaction paradigms is the fact that, once an experimental variation has been introduced, it most likely develops its own dynamics (Bente and Krämer, 2011).

One of the most promising approaches to study non-verbal communication in “online” social interactions is the “transformed social interaction” (TSI) approach (Bailenson et al., 2004; Krämer, 2008), which builds upon the previously mentioned “plasticity” advantage of VCs (Bente et al., 2008). In this approach, motion is captured and rendered on an avatar. Not only the appearance of the VC (containing information for instance on sex, identity, ethnicity, or attractiveness), but also their non-verbal behavior can be manipulated, by blending particular channels (via static filters), or by modifying specific non-verbal cues (via dynamic filters, e.g., head movement activity can be altered using specific algorithms) (Bente and Krämer, 2011). By doing this in a systematic manner, it can be determined which aspects of non-verbal behavior are necessary and/or most efficient with regard to various social contexts. This makes it possible to analyze how manipulations of appearance and/or behavior of one agent or a dyad affect the experience and the course of social interactions. Blascovich et al. (2002) (p.121) summarize the benefits of this approach by stating “investigators can take apart the very fabric of social interaction using immersive virtual environment technology (IVET), disabling or altering the operation of its components, and thereby reverse engineering social interaction. With this approach, social psychologists could systematically determine the critical aspects of successful and unsuccessful social interactions, at least within specified domains and interaction tasks.”

The TSI approach has been used to study the effects of experimentally manipulated gaze behavior in ongoing interactions. Bente et al. (2007a,b), used eye tracking and motion capture to control two avatars representing two interactants during an open conversation. While gestures and movements were conveyed in real time, the display of gaze direction was manipulated. The authors could show that longer periods of directed gaze fostered the positive evaluation of the partner. The study demonstrated how experimental control of non-verbal cues can be implemented within a rich and fluent social interaction. Other studies using gaze-contingent eye-tracking paradigms have been developed to investigate how social gaze is used to coordinate attention between a participant and an agent (Schilbach et al., 2010; Wilms et al., 2010; Pfeiffer et al., 2011). Finally, paradigms investigating the social effects of mimicry during social interactions with VCs have been developed as well (Bailenson and Yee, 2005, 2007).

To conclude, the “online” social interaction approach in non-verbal behavior research asks for the analysis of behavioral as well as physiological and neural patterns emerging across agents during social interactions. The specific advantages of using VCs in this type of research have been demonstrated for rather simple and restricted non-verbal cue systems, such as social gaze. Nevertheless, this approach can be easily extended to higher complexity levels in non-verbal behavior (see also “shared virtual environment” in Figure 1).

### Recent developments in neuroimaging paradigms using virtual characters as real-time interaction partners

Using VR in neuroimaging paradigms [for instance functional magnetic resonance imaging, functional magnetic resonance imaging (fMRI) paradigms] increases the potential of standard fMRI paradigms, where the volunteer usually has the passive role of watching a simple stimulus without any interaction. In this line, De Gelder and Hortensius (2014) (p. 160) argue that VR provides the field of social-cognitive neuroscience with a powerful tool to study affective loops created by “online” interactions “in settings where real-life manipulation is not possible, too expensive or unethical.”

In neuroimaging studies, however, despite the technical advancement of the VR and motion capture technologies, the possibilities of studying non-verbal communication in social interaction are limited. First, social scripts would have to be systematically manipulated, reduced, and presented repeatedly, in order to increase statistical power. Moreover, not only need the experimenters ensure that the motion tracking systems are compatible with the available neuroimaging techniques (e.g., MR-compatible for fMRI experiments), but participants are also restricted in their movements to prevent causing artifacts during neural data acquisition. Indeed, research is being done on developing VR platforms that are compatible with magnetic resonance imaging systems (e.g., Baumann et al., 2003; Mraz et al., 2003; see also Figure 1).

There are, however, several ways to overcome this problem. One way would be to create VEs, where the visual embodiment of the participants and hence their means of interaction with the virtual world and virtual other is controlled via some limited input information in the scanner environment. Although far from ideal (since it involves awareness and explicit production of non-verbal behavior), this approach presupposes that a virtual avatar can be controlled by using button presses or a joystick. In this line, Baumann et al. (2003) have developed a VR system of integrated software and hardware for neurobehavioral and clinical studies for fMRI studies. The authors propose a VR system, which includes a joystick for navigation, a touchpad, and an optional data glove with an attached motion tracker. Furthermore, the setup enables the measurement of physiological data (respiration, heart rate, blood volume pulsatility, and skin conductance response), and the system provides synchronization of the VR simulation with the physiological recordings and the functional MR images (see also “brain imaging setups” in Figure 1).

Another possibility of using VR in the fMRI context would be to investigate a form of minimal social interaction, which would, by definition, only require minimal non-verbal input but would enable the study of social interactions based on gaze behavior in real time. In this line, social gaze paradigms offer a good solution (Schilbach et al., 2010; Wilms et al., 2010; Pfeiffer et al., 2011, 2013b). Recent methodological advances have used VCs [for a review see Barisic et al. (2013)] in (1) gaze-contingent eye-tracking paradigms (Schilbach et al., 2010; Wilms et al., 2010; Pfeiffer et al., 2011; Grynszpan et al., 2012), (2) live interactions via video feeds, as in bi-directional real-time video streams (Redcay et al., 2010; Saito et al., 2010; Tanabe et al., 2012), and (3) dual eye tracking in two (real or virtual) person setups (Barisic et al., 2013). In the first approach, the gaze is used to control contingent behavior of VCs, who are agents with preprogrammed reactions contingent upon an individual’s behavior, hence creating merely the illusion of an “online” real-time interaction (Barisic et al., 2013). The second approach does not make use of virtual technology; therefore, the experimenter is unable to interfere with an interaction, except for substituting or delaying the real-time video stream (Barisic et al., 2013). Consequently, only the third approach is a real interactive one, able to make full use of avatar technology. In this line, Barisic et al. (2013) present the implementation of a dual eye-tracking setup enabling true reciprocity and coordination in a social interaction of two individuals represented by avatars. In this setup, the eye gaze can be either an active part of the task or it can be a dependent measure that can be correlated with other behaviors of interest (Barisic et al., 2013). Furthermore, in line with the TSI approach, this paradigm allows both the VE and the VCs to be fully and systematically controlled in terms of their outer appearance and behavior.

A further promising approach in terms of neuroimaging possibilities, which open up another level of analysis in this line of paradigms, is hyperscanning. It allows the simultaneous measurement of brain activity in two interacting individuals situated in different neuroimaging environments (electroencephalography: Astolfi et al., 2010; Dumas et al., 2010; Kourtis et al., 2010; Lachat et al., 2012; near-infrared spectroscopy: Cui et al., 2012; magnetoencephalography: Baess et al., 2012; Hirata et al., 2014; fMRI: Montague et al., 2002; King-Casas et al., 2005; Saito et al., 2010; Tanabe et al., 2012). In particular, the development of fMRI hyperscanning allows the synchronization of functional image acquisition across multiple subjects and scanners, the performance of cross-brain correlation analyses and, thus, permits the measurement of inter-brain activity coherence during the act of interacting. Combined with using VCs as stimuli, hyperscanning would allow researchers to measure the reactions of multiple participants to shared social situation in a VR environment (see also “Virtual characters as real-time interaction partners” and “brain imaging setups” in Figure 1).

### Virtual characters and social presence

The acceptance of VCs as intentional and engaging social entities has also been described as “copresence” (also referred to as “social presence”) to describe a communicator’s sense of awareness of the presence of an interaction partner [for a review, see Biocca et al. (2003)]. While we can conclude that numerous studies by different research groups show that people can perceive both forms of representations (agents or avatars) comparably to real human beings, it is important to note that findings are not entirely conclusive (cf. Perani et al., 2001; Mar et al., 2007; Moser et al., 2007). The emergence of copresence, which can be measured both at the behavioral and neural level, is mediated by several factors, and the “immersion” potential of the technology is only one of them. Such factors need to be taken into account when designing social interaction paradigms using VCs. In the following, these factors are described.

#### Anthropomorphism (i.e., human form realism)

The consensus in the literature argues that the more anthropomorphic or humanlike a character looks like, the more likely they are accepted by an observer (Garau, 2003). On a neural level, the activity of neural regions of the SNN, which are consistently associated with social-cognitive processing, is correlated with the increasing degree of realism of a character (Mar et al., 2007), or anthropomorphism of an interaction partner while performing the prisoner’s dilemma game (Krach et al., 2008). In a similar vein, it has been suggested that the AON is tuned to realistic representations of conspecifics (Perani et al., 2001; Shimada, 2010).

#### Behavioral realism (i.e., humanlike movements or behavior patterns)

In observational paradigms, the AON has been found to be preferentially activated when processing biological motion, i.e., movements with kinematics characterized by a smooth velocity profile [Dayan et al., 2007; Casile et al., 2010; but see also Cross et al. (2012) and Georgescu et al. (2014)]. In interactive paradigms, aspects of behavioral realism related to the responsiveness of or feedback from the VC are crucial for the emergence of social presence (Garau, 2003). Indeed, even subtle manipulations increasing an avatar’s responsiveness (e.g., maintaining eye contact, realistic blinking rates) can influence participant’s social responses to VCs suggesting that on some levels people can respond to virtual humans as social entities even in the absence of complex interactions (Bailenson et al., 2001, 2002; Garau et al., 2005).

#### The interaction between anthropomorphism and behavioral realism and the “uncanny valley” effect

Generally, it has been suggested that the sensitivity to biological motion is independent of how detailed the character’s body is (Chaminade et al., 2007; McDonnell et al., 2008). However, according to the “uncanny valley” theory, the more a VC looks like a real human, the more likely subtle imperfections are perceived as awkward and therefore allocate attention to other processes than the targeted social-cognitive processes (Mori, 1970; Garau, 2003). Hence, even subtle flaws in rendering or expression may cause irritations when extremely detailed anthropomorphic, fully rendered 3D characters are used. In this regard, VCs that are highly realistic might set up high expectations also with respect to behavior realism. Hence, a mismatch between form and behavioral realism can lead to a perception of inconsistency (Garau, 2003; Nowak and Biocca, 2003; Saygin et al., 2011). Indeed, even the most advanced motion capture technologies may find it impossible to match the level of accuracy in terms of degrees of freedom of natural human movement.

#### Agency (the belief or knowledge about the nature of a VC)

A top-down influence of belief about the nature of the VC (agent or avatar) may also modulate its perception [Stanley et al., 2007, 2010; Liepelt and Brass, 2010; Klapper et al., 2014; but see Press et al. (2006) and von der Pütten et al. (2010b)]. A direct comparison between the influence of belief about agency and behavioral realism on social presence revealed that believing to interact with an avatar or with an agent barely influenced the evaluation of the VC or his behavioral reactions, whereas variations in behavioral realism affected both [von der Pütten et al., 2010b; see also Nass and Moon (2000)].

#### Observer characteristics

It is important to note that, while VCs can elicit social presence, they tend to do so to varying degrees in different observers. Certain characteristics of the observers such as age and sex, their perceptual, cognitive and motor abilities, or prior experience with mediated environment can influence the amount of experienced social presence. For instance, participants’ subjective feeling after an interaction with an embodied conversational agent, as well as their evaluation of the VC and their actual behavior was dependent upon their personality traits (von der Pütten et al., 2010a). Furthermore, an important factor to control for is the computer proficiency of the observers and their exposure to VCs. Some people have a higher affinity to computers and games that use avatars and may thus have different expectations concerning both form and behavioral realism. There is evidence that proficiency in using VEs, facilitates immersion and/or copresence, and training with artificial human stimuli can increase their credibility (Garau et al., 2005; Press et al., 2007). Similarly, Dyck et al. (2008) found that emotion recognition rates decreased for virtual but not for real faces only in participants over the age of 40, indicating that media exposure may indeed have an influence on the recognition of non-verbal signals displayed by VCs.

## Virtual Characters in Non-Verbal Behavior Research in HFA

### Virtual characters as stimuli in neuroimaging studies

A critical prerequisite for a reasonable use of VCs in research investigating non-verbal behavior processing in HFA is that autistic individuals are engaged by VCs to the same extent as they would be by real human beings and that they do not show any differential positive or negative psychological responses to the former. Hernandez et al. (2009) performed an eye-tracking study to quantify gaze behavior in both adults with ASD and typically developing individuals while exploring static real and virtual faces with direct gaze. In concordance with the literature, participants with HFA spent less time on the eye region compared to typically developing individuals (e.g., Pelphrey et al., 2002; Rutherford and Towns, 2008; Riby and Doherty, 2009; Nakano et al., 2010; Falkmer et al., 2011). Critically, no differences were identified with regard to the exploration of the faces depending on whether they were real or virtual. With respect to the experience in IVEs, Wallace et al. (2010) have for instance shown in a usability study on HFA and typically developing children that experience of IVEs was similar across groups, and no negative sensory experiences were reported in children with HFA. We can conclude that VCs and IVEs are experienced in a similar manner by individuals with HFA and typically developing individuals, and that they can reliably be used to simulate authentic social situations in experimental settings. To our knowledge only five neuroimaging studies on non-verbal cue processing have been performed on individuals with HFA. In the following, we will review this literature (see also Table 1 for an overview and details of the paradigms).

**Table 1:** **Overview of neuroimaging studies using virtual characters to study non-verbal behavior processing in HFA**.

Study	Participant characteristics	Experimental design and stimuli	Description of results
Pelphrey et al. (2005) fMRI Eye gaze direction (congruency)	*N* = 10 ASD (mean age: 23.2), right-handed; compared to other studies conducted in his laboratory	Stimuli: short videos of a VC shifting their gaze either congruently or incongruently with the location of an appearing checkerboard Design: 10 runs (70 trials of each condition); eye tracking Task: attend to the screen at all times, but allowed to look at presented stimulus in any way the wish; press a button with right or left thumb when eye movement is seen, no matter whether the eyes acquire the target	Behavioral: Eye tracking showed no differences between subjects with and without ASD Neural: Subjects in autism do not show differences in activity of the STS and other brain regions linked to social cognitions Activity in these regions was not modulated by the context of the perceived gaze shift
Pitskel et al. (2011) fMRI Eye gaze direction (direct vs. averted)	*N* = 15 male HFA (mean age: 23.4) and 14 matched controls (mean age 24.2)	Stimuli: an approaching male VC maintained either direct or averted gaze with the observer Design: one run (422 s), 10 trials (6 s per trial) intertrial intervals of 12, 14, or 16 s Task: attend to the displays and remain alert and awake	Behavioral: Both participant groups were sensitive to the experimental manipulation, yet the gaze condition that elicits preferential neural activation differs as a function of group status Neural: TD: greater activation to direct gaze in the right anterior insula (AI), bilateral caudate, left thalamus, left cerebellum, and left inferior frontal gyrus HFA: greater activation to direct gaze in left cuneus, and greater activation to averted gaze in bilateral cerebellum and left inferior occipital gyrus. No correlations between activation in regions modulated by gaze condition correlated significantly with age or Full Scale IQ
			Right AI only showed significant differences between gaze in the typically developing group, while left LOC was only significantly modulated by gaze in the autism group
Schulte-Rüther et al. (2011) fMRI Emotional facial expression recognition	*N* = 18 male ASD (mean age: 27.40) and 18 matched controls; only 14 of each group included in final fMRI (ASD group: 7 AS, 7 HFA)	Stimuli: three dimensional representations made of male faces, morphed to happy or sad expression (each with how or low intensity) or neutral expression (2 × 2 × 2 and 3 × 2); static Design: block design; 12 blocks with three experimental tasks, each block consisting of 6 trials (total of 192 trials) Task: identify emotional expression of face (other) or emotion elicited in themselves by the face (self)	Behavioral: Reaction times were faster for the other- than for the self-task and faster for the high than the low emotion intensity stimuli Number of correct responses for the other-task was higher than the number of congruent responses for the self-task and higher for the high emotional intensity than the low emotional intensity stimuli Neural: Other-task vs. control task: control subjects showed differential activation in the vMPFC and precuneus/PCC, subjects with autism showed differential activation in the dMPFC Self-task vs. control task: control subjects showed additional activations in the dMPFC, left IFC, left TPJ, and right Cerebellum, subjects with ASD showed increases in activity of left superior frontal gyrus, bilateral middle frontal gyrus, bilateral IFC, bilateral TPJ, ITG and temporal pole Conjunction of other task vs. control task and self-task vs. control task: in ASD subjects, conjoint activation could be observed in bilateral precuneus/PCC and left dMPFC
Georgescu et al. (2013) fMRI, eye tracking Eye gaze direction (direct vs. averted) and duration	*N* = 13 HFA (9 male, mean age:31.23) and 13 matched controls (9 male, mean age: 30.23)	Stimuli: ca. 5 s animations of 10 male and 10 female VC neutral faces, displaying either averted or direct gaze of varying duration (1, 2.5, 4 s) Design: 2 × 3, parametric; event-related; factor (1) gaze direction (direct and averted) and factor (2) gaze duration (1, 2.5, 4 s) Task: judging likeability of each VC on a 4-point scale	Behavioral: HFA participants showed no significant difference in likeability ratings depending on gaze duration Control group rated the virtual characters as increasingly likeable with increasing gaze duration No significant group difference Eye tracking: no difference in fixation on any face ROI across conditions and across groups Neural: In controls: Regions of the SNN are activated by direct vs. averted gaze and by increasing gaze duration perception In HFA: the pSTS is activated by direct compared to averted gaze; no differential activation for processing increasing gaze duration: regions of the SNN are engaged by averted compared to direct gaze and by decreasing gaze duration
von dem Hagen et al. (2013) fMRI, eye tracking Eye gaze direction (direct vs. averted)	*N* = 21 male HFA and AS (mean age: 29) and 25 matched controls (mean age: 26)	Stimuli: animations of 5 male and 5 female VC neutral faces, displaying either averted or direct gaze Design: block design 21-s long epochs Task: gender judgments	Neural: No group differences in DMNb, DMNc, salience, and MTL networks within or without ROIs Significantly reduced functional connectivity between and within resting state networks

Neuroimaging research investigating non-verbal behavior in HFA using VCs has focused mainly on face processing and more specifically on the processing of emotional expressions and eye gaze. Schulte-Rüther et al. (2011) asked participants to empathize with static virtual emotional faces and either judge the emotional state of the face (“other” condition) or report the emotions elicited in themselves by the emotional face (“self” condition). With respect to the behavioral performance, the authors found no significant differences in reaction times between HFA participants and control participants for any of the two experimental conditions. In addition, neural results showed that key areas of the SNN were activated in both controls and HFA participants. However, the authors found evidence for a functional segregation in the medial prefrontal cortex, a region which has previously been associated with mentalizing (Amodio and Frith, 2006). Direct comparisons showed that the self- and other-referential tasks, relative to the control task, engaged the dorsal medial prefrontal cortex for individuals with HFA and the ventral portion of the same region for control participants. According to the established functional characterizations of these neural regions, empathizing with other persons is likely to be triggered by emotional self-referential cognition in controls, as affective “theory of mind” components are known to recruit ventral areas of the medial prefrontal cortex region (Amodio and Frith, 2006). Conversely, HFA participants seem to engage cognitive components of “theory of mind,” which are associated with the dorsal portion of the medial prefrontal cortex (Amodio and Frith, 2006).

Given that eye gaze provides a foundation for communication and social interaction (Senju and Johnson, 2009), one area of research that has particularly benefited from the use of VR techniques has been concerned with investigating the neural correlates of social gaze processing in HFA. One of the first neuroimaging studies on this subject used fMRI to show that, in HFA, brain regions involved in gaze processing are not sensitive to intentions conveyed by observed gaze shifts (Pelphrey et al., 2005). The paradigm was based on short videos of a VC shifting their gaze either “congruently” at a checkerboard that appeared in their visual field, or “incongruently” away from the checkerboard. Autistic participants engaged the same temporo-parietal network as controls to process this task, which was centered around the superior temporal sulcus. However, in contrast to the control group, their activation was not modulated by congruency. The authors suggest that an absence of contextual influence on the superior temporal sulcus region indicates a reduced understanding of different intentions of others’ gaze behavior and may represent a possible mechanism underlying gaze-processing deficits reported in ASD. More recently, Pitskel et al. (2011) addressed the differential neural processing of direct and averted gaze. Participants viewed videos depicting an approaching male VC either maintaining direct gaze with the observer or averting their eyes from them. The SNN, which was more responsive to the direct relative to averted gaze in typically developing participants, was not preferentially active to direct gaze in HFA participants, indicating again a reduced understanding of different meanings of non-verbal social cues. Similarly, von dem Hagen et al. (2013) showed participants dynamic virtual faces with neutral expressions displaying either averted or direct gaze events. Their results showed that regions of the SNN were more involved in processing direct compared to averted gaze in control participants but in the opposite contrast for HFA participants, potentially indicating an increased salience of averted but not direct gaze in HFA. Finally, a study from our own research group (Georgescu et al., 2013) employed a parametric design in order to investigate the neural correlates of the influence of gaze direction and duration on person perception. We used dynamically animated faces of VCs, displaying averted gaze or direct gaze of varying durations (1, 2.5, or 4 s). Results showed that direct gaze as such and increasing direct gaze duration modulated the engagement of the SNN in control participants, indicating the processing of social salience and a perceived communicative intent. In HFA participants, however, regions of the SNN were more engaged by averted and decreasing amounts of gaze, while the neural response for processing increasing direct gaze in HFA was not suggestive of any social information processing.

To conclude, research using VCs as stimuli attests that they are a useful tool for the investigation of non-verbal behavior processing in HFA. In particular, the inclusion and manipulation of dynamic aspects of movement is facilitated by using VCs and is therefore able to offer unique insight into non-verbal behavior processing in HFA. As a general result, these research findings show atypical social-cognitive processing in HFA both on the behavioral and neural level, highlighting the fact that non-verbal information is less salient to individuals with HFA compared to typically developed individuals. But while the mere perception of non-verbal cues may, under certain circumstances, be comparable to that of typically developed individuals, it seems that in individuals with HFA the evaluation of such cues may rely on different cognitive strategies.

### Virtual characters as real-time interaction partners

Despite the strong evidence for social processing deficits in HFA individuals, it has been documented that persons with HFA may learn to compensate their performance during social situations in structured experimental settings (Kylliäinen and Hietanen, 2004; Congiu et al., 2010). Such explicit instructions to focus on specific social contents of stimuli may cancel out the atypical performance effects and even diminish the typical hypoactivation of SNN areas (Wang et al., 2007; Schulte-Rüther et al., 2011). In a similar vein, anecdotal reports inform us that individuals with autism have particular problems during “online,” real-time interactions, which require the integration of signals from a variety of channels, while the complex and unpredictable input is rapidly changing (Redcay et al., 2012; Wang and Hamilton, 2012). This is in line with the idea that ASD is a disorder of complex information processing (Minshew and Goldstein, 1998). Dynamic interactions critically impede the application of rule-based strategies to compensate for HFA-characteristic deficits in intuitive communication (Klin et al., 2003; Redcay et al., 2010). This points to the fact that “offline” social cognition paradigms as the ones described above (in “Virtual characters as stimuli in neuroimaging studies”), may fail to capture important aspects of social processing deficits in HFA, and that “online” paradigms might be more appropriate for this purpose. Thus, neuroimaging paradigms using VEs and VCs that include the complexity of dynamic social interactions, may provide a more sensitive measure of the neural basis of social and communicative impairments in HFA. Consequently, the combined use of VR and neuroimaging techniques offers great potential to investigate non-verbal communication in social interactions as well. Therefore, the possibility of engaging HFA in interactions in the scanner may be useful in understanding social cognition in ASD (Redcay et al., 2010).

Social interactions are characterized by a high degree of automatic interpersonal coordination (Cappella, 1981, 1996; Burgoon et al., 1993) or an above-chance probabilistic relationship between the actions of two interactants (Moran et al., 1992). A number of studies have performed kinematic analyses to investigate motor patterns expressed in social interactions and were able to show that the kinematics of an action performed by an agent acting in isolation are different from those of the very same action performed within a social communicative context and that kinematics not only carry information as to whether a social communicative action is performed in a cooperative or competitive context, but also that they cause flexible online adjustments to take place in response to a partner’s actions [Georgiou et al., 2007; Becchio et al., 2008a,b; Sartori et al., 2009; for a review, see Becchio et al. (2010)]. The ability to detect an interaction partner’s responses as being related to one’s own is termed social contingency sensitivity (Bigelow and Rochat, 2006). Several studies using observational paradigms have already attested that individuals with ASD perceive contingency in dyadic interactions abnormally in terms of animacy perception and mental state attribution [Abell et al., 2000; Klin, 2000; Castelli et al., 2002; Klin and Jones, 2006; see also Centelles et al. (2013)]. Therefore, it has been hypothesized that this inability to detect contingency in social interactions (as either observers or participants), may be a core impairment in autism (Gergely, 2001). The inefficient contingency processing could be related to one particular non-diagnostic secondary symptom of HFA, namely atypical temporal processing. For instance, interval timing [i.e., processing of stimulus duration; for a review, see Falter and Noreika (2011); see also Georgescu et al. (2013) for duration processing for social cognition] and temporal event structure coding (Falter et al., 2012, 2013) have been found to be atypical in HFA. There is evidence for the association between temporal processing and social cognition (e.g., Moran et al., 1992; Trevarthen and Daniel, 2005; Bigelow and Rochat, 2006), Thus, atypical temporal processing might play an important yet under-investigated role in ASD by interacting with and modulating primary symptoms, like deficits in non-verbal communication and social coordination and interaction (Falter and Noreika, 2011).

One such aspect that depends on temporal processing in the social domain is motor mimicry. Elementary motor mimicry (i.e., when an observer’s overt motor response is appropriated to the situation of the observed other) has been understood as a communicative act (Bavelas et al., 1986). Bailenson and Yee (2005) performed the first study to show social influence effects with a non-human, non-verbal mimicker (i.e., an imitator of the behavior of another; Chartrand and Bargh, 1999). The authors found that when an embodied virtual agent mimicked participants’ head movements 4 s after they occurred during a social interaction, the mimicking agent was more persuasive and was rated more positively with respect to certain traits compared to non-mimickers. The STORM (i.e., “social top-down response modulation”) model of mimicry claims that mimicry is socially top-down modulated and subtly controlled by social goals (Wang and Hamilton, 2012). For example, in typically developed individuals, social context as communicated through eye contact has been found to control mimicry by modulating the connection strength from the medial prefrontal cortex, a key region of the SNN, to regions of the AON (Wang et al., 2011). Future studies are currently being planned that will examine how different types of social information and social goals are used in the control of mimicry and whether the mimicry production and processing is abnormal in HFA (Wang and Hamilton, 2012). One particularly promising approach in this respect would be the TSI approach, similar to the one used by Bailenson and Yee (2005). This could involve creating VCs that can copy a participant by using an automatic mimicking (i.e., a computer algorithm applied to all movements), to test how people with HFA respond to and detect different social cues from the VC.

Hyperscanning (described in “Virtual characters and social presence”) may be combined with experimental paradigms to characterize the neural dynamics contributing to atypical social processing in HFA (see Figure 1 for a possible setup involving eye tracking and manual response options). For instance, a recent fMRI hyperscanning study in which dyads either comprising a participant with ASD and a control participant or two control participants engaged in a gaze- vs. target cued joint attention task (Tanabe et al., 2012). Among other findings, the authors report a reduction of inter-individual coherence of intrinsic activity fluctuations in ASD-control as compared to control-control pairs in the right inferior frontal gyrus. The authors speculate that this finding might be related to decreased motor resonance for gaze behavior in these dyads.

## Virtual Characters as a Supportive, Educational, and Therapeutic Tool for HFA

While the majority of adults with HFA consider the access to assistive therapy options as an important issue (Gawronski et al., 2011), many individuals have great difficulty finding traditional trainings and intervention approaches due to intervention costs and a lack of available specialized therapists (Bekele et al., 2013). In this line, the use of VEs and VCs offers an alternative, which could increase intervention accessibility and reduce the cost of treatment (Goodwin and Goodwin, 2008).

By enabling the simulation of a social environment, VR provides opportunities to practice dynamic and real-life social interactions in a safe environment (Krämer, 2008). Furthermore, VR possesses several advantages in terms of potential application for individuals with HFA. These are listed in the following, and while they represent independent features of VR technology, it is their combined value that offers unique potential for individuals with HFA (Parsons and Cobb, 2011).

### Control

The level and number of various features of the environment can be directly controlled and manipulated. This enables frequent practice and/or exposure in a variety of repeatable and adjustable situations that mimic the real world (Krämer, 2008).

### Flexibility

Increased control over the scenarios and environments allows for interfaces to be modified for individual user needs, for intervention approaches and reinforcement strategies, as well as scenarios to be customized (Strickland, 1997; Rizzo and Kim, 2005; Krämer, 2008):

### Error-free learning

Increased control also allows for competing or distracting stimuli to be removed from the training setting and the level of exposure to be carefully controlled (Strickland, 1997; Parsons and Mitchell, 2002; Rizzo and Kim, 2005; Krämer, 2008). Users’ performance can be recorded and used for subsequent discussion. Thus, users can practice without fear of mistakes or rejection (Rizzo and Kim, 2005; Krämer, 2008).

### Independent practice

Self-guided exploration and independent practice in a safe test/training environment are enabled (Rizzo and Kim, 2005), where the user has active control over their participation (Parsons and Mitchell, 2002).

### Ecological validity

This offers greater potential for naturalistic performance measures with real-time performance feedback, hence increasing the potential for generalization (Parsons and Mitchell, 2002; Rizzo and Kim, 2005; Bellani et al., 2011; Wang and Reid, 2011).

### Affinity with computers

Finally, this approach can be particularly useful for persons with ASD, as they have been found to have a natural interest in and affinity with computers due to the predictable, consistent, and repeatable nature of technology (Parsons and Mitchell, 2002; Parsons et al., 2006; Putnam and Chong, 2008). This, in turn, could heighten their compliance and investment in the treatment (Krämer, 2008) and may be even made use of, for example, by including gaming factors to enhance user motivation to complete tasks (Rizzo and Kim, 2005).

Indeed, usability research has attested participants’ explicit acknowledgment of the value of the virtual training for them (e.g., Parsons and Mitchell, 2002; Parsons et al., 2006). Research has also shown that individuals with ASD successfully acquire new information from VEs. In particular, they learn how to use the equipment quickly and show significant improvements in performance after training [for reviews, see Strickland (1997), Bellani et al. (2011), Parsons and Cobb (2011, 2013), and Wang and Reid (2011)]. Some authors have investigated the usefulness of VEs for training behaviors such as crossing the road (Josman et al., 2008) or reacting to a tornado warning (Self et al., 2007) and to aid learning of pretend play (Herrera et al., 2008). However, we will focus the following considerations on virtual training and assistive therapies on the advantage of VEs for training social and non-verbal skills.

Virtual characters have been used for individuals with HFA to provide training to teach social conventions, facilitate acquisition and exploration of social skills, and reduce stress in social situations. For instance, some social skill training scenarios involve finding a place to sit in a crowded canteen, cafe or bus, a job interview or shopping situation (Rutten et al., 2003; Parsons et al., 2006; Mitchell et al., 2007), or training collaboration skills in the context of the production of a joint narrative (Gal et al., 2009). Jarrold et al. (2013) have developed a public speaking task using IVE technology to study social attention in HFA. They used a HMD to display a virtual classroom and assess the ability of children with HFA to answer questions and simultaneously attend to nine avatar peers seated at a table. They have found that HFA, compared to controls, looked less frequently to avatar peers in the classroom while talking. Consequently, in order to train social attention, virtual training programs have been developed (Grynszpan et al., 2009; Lahiri et al., 2011a,b). For example, Lahiri et al. (2011a,b) developed a novel paradigm, able to automatically structure and adapt interactions in real-time. The platform is called the “Virtual Interactive system with Gaze-sensitive Adaptive Response Technology” (VIGART) and is capable of monitoring a user’s gaze in real-time and delivering individualized feedback based on the user’s dynamic gaze patterns during their interaction with a virtual other. The experimental setup involved a DVE that presented participants with social communication tasks. While the participant viewed the avatar narrating a personal story, the participant’s viewing patterns were measured in real-time by acquiring gaze data and subsequently some behavioral viewing indices were computed. These episodes were followed by a short quiz on the content of the virtual other’s personal story. After the participant’s reply, an audio-visual feedback, which was computed based on the real-time gaze data to determine the actual time the participant spent looking at the face of the avatar during the presentation, was provided to the participant. The idea was to give indirect feedback to the participants about their viewing patterns and thereby study how that would affect the participants as the task proceeded. Preliminary data for six adolescents with ASD indicate improvement in behavioral viewing and changes in relevant eye physiological indexes of participants. Another approach was introduced by Porayska-Pomsta et al. (2012) who developed the ECHOES project. It aims to allow children with social difficulties to understand and explore social communication and interaction skills. In this learning platform, children interact with embodied virtual agents in socially realistic situations. The interaction between the child and the agents is facilitated by a combination of learning activities, designed around specific learning goals that relate to different forms of joint attention and turn-taking as well as free exploration of the environment.

The environments used in such approaches have been either single-user virtual environments (SVEs) or collaborative virtual environments (CVEs). In an SVE, a single user explores the VE and responses from the environment or a virtual agent must be preprogrammed. In a CVE, more than one user may inhabit the VE at the same time (for example the patient and the therapist or trainer) and can interact with each other in real-time via avatars. Users control their avatars independently and can communicate directly with each other, even when physically located in different places, through speech, movement, and gesture in the virtual space (Schroeder, 2002; Rutten et al., 2003; Moore et al., 2005; Bellani et al., 2011; Millen et al., 2011; Parsons and Cobb, 2013). For example, the COSPATIAL project developed and evaluated collaborative technologies for engaging children with autism in social communication, involving perspective-taking, conversation, and collaboration games (Millen et al., 2011). Rutten et al. (2003) performed a usability study to investigate the potential of CVEs for individuals with ASD. They used role-play situations in either a meeting room or a social cafe. Although the emphasis lied on verbal communication between avatars, users were able to activate some basic non-verbal signals like a handshake and a smiling, neutral or frowning facial expression. The authors conclude that CVEs provide less inherent structure than SVEs and more scaffolding of learning is usually required to keep interactions flowing. Nevertheless, they offer increased flexibility for training in social skills, which do not rely on a fixed protocol hence providing opportunities for social skill practice in a less structured, yet more naturalistic and ecologically valid manner (Rutten et al., 2003). The authors suggest that the most productive setup in communication outcome for a CVE would be when a teacher or trainer supports the users, and a confederate plays the role of another avatar. In a similar line, Parsons et al. (2006) argue that the so-called “facilitators” in a training or intervention are an essential part of the learning process, helping the user interpret what is happening in the scene, take another’s perspective and make appropriate responses accordingly. The role of the facilitator should always be adequately planned and provided for as an integral design feature of VEs for teaching of social skills (Parsons et al., 2006). Given that ASD have been associated with executive dysfunction (Hill, 2004) and that complexity in terms of task demands and sensory input information may be challenging for autistic individuals (Minshew and Goldstein, 1998; Redcay et al., 2012), it is essential to invest into optimal design research for platforms and software targeted at HFA individuals (Grynszpan et al., 2005, 2008; Wallace et al., 2010; Menzies, 2011). In this line, transformed virtual interactions might be a promising approach (Bailenson et al., 2004). Tracking non-verbal signals and rendering them via avatars allows for a strategic decoupling of communication (Bailenson et al., 2004), which would allow to alter the exchanged information between sender and receiver and increase or decrease gradually the level of complexity of the social situation, while still facilitating error-free learning.

Collaborative virtual environments also bear advantages in terms of non-verbal decoding and encoding skills. In the non-verbal domain, CVEs have been used to examine and investigate the ability to recognize emotions (Moore et al., 2005; Fabri et al., 2007) and also teaching students how to manifest their emotions and understand those of other people (Cheng and Ye, 2010). These studies found a good performance in identifying emotions and an improvement in social performance after the intervention. Krämer (2008) suggests three important requirements for a non-verbal skills training: (1) realistic setting that requires both decoding and encoding of non-verbal cues; (2) immediate non-verbal feedback from the interaction partner; and (3) feedback that is given not only with regard to demonstrative cues of the user, but also with regard to subtle aspects of their behavior, like the movement speed or quality. Indeed, VEs and virtual training partners seem to allow the development of training paradigms that fulfill these criteria.

While most training approaches using VCs and VEs have been developed for children and adolescents, Kandalaft et al. (2013) have developed the first Virtual Reality Social Cognition Training (VR-SCT), targeted at the adult HFA population. The intervention is using a CVE paradigm and a DVE setup and focuses on enhancing social skills, social cognition and social functioning. Its feasibility was tested on a group of eight HFA adults, who completed a total of 10 sessions across 5 weeks. Results showed significant increases on social-cognitive measures of theory of mind and emotion recognition, as well as in real-life social and occupational functioning.

Indeed, the literature is increasingly recognizing the potential benefits of VR in supporting the learning process, particularly related to social situations, mostly in children and adolescents with autism [for reviews, see Strickland (1997), Bellani et al. (2011), and Parsons and Cobb (2011)] and also in adults (Kandalaft et al., 2013). Nevertheless, some challenges need to be mentioned as well. Current approaches only involve small samples and more randomized controlled trials with treatment manipulations and matched control groups need to be performed in order to show the effectiveness of a certain training and whether the improvements can transfer to real-life situations. Moreover, while virtual technologies are rapidly advancing, developing training or therapeutic tools using VCs and VEs involve a great amount of time, effort, and resources, as well as a multidisciplinary dialog.

## Conclusion

In conclusion, we have argued that the use of VCs can be of great value for experimental paradigms of social cognition, in particular for such paradigms concerned with non-verbal behavior production and perception. There are several points to note with respect to challenges inherent in the use of VCs and VEs. First, the compromise or tradeoff between ecological validity and experimental control constitutes both an advantage and a limitation of the approach. Second, individuals may have varying degrees of exposure to or experience with VCs, which may influence their expectations during observation of or interaction with them. Third, different age groups may also react differently to the stimuli and settings and may require different tasks and social situations to be implemented. Furthermore, limitations may also arise from the time and effort that needs to be invested in developing virtual and neuroimaging technologies. In a similar line, these developments need to take place in the context of multidisciplinary research endeavors, which brings an interesting set of challenges on its own (i.e., fruitful collaboration and communication of experts across disciplines). On a positive note, De Gelder and Hortensius (2014) summarize that the use of VR will give the field of affective and social neuroscience valuable and important tools to grasp the full extent of the social world in a well-controlled manner. We have argued that artificial humans are a useful and valid tool to overcome common methodological problems in non-verbal behavior research and may offer an efficient solution for the development of real-time “online” social interaction studies using the TSI approach. This would potentially allow to “reverse engineer” social cognition (Blascovich et al., 2002), by enabling a detailed and systematic examination of the contribution of various real-time factors in human social interaction. Moreover, not only can VCs inform us about human social cognition, both typical and atypical, but they can also contribute to the development of design and methodology for creating interactive agents (Vogeley and Bente, 2010). We have also argued that VCs and environments are a valuable tool for the supportive therapies and the training of social skills and non-verbal decoding in HFA, as they provide a safe, repeatable and diversifiable learning environment. In addition, the growing trend toward CVE setups becomes evident for therapeutic technologies as well. The methodology for designing interactive multimodal technology for autistic persons requires extensive research and multidisciplinary expertise including developmental psychology, visual arts, human–computer interaction, artificial intelligence, education (Porayska-Pomsta et al., 2012). Future research can additionally investigate how newly acquired skills trough such training programs are transferred to the real world and describe their impact on a neural level (Bellani et al., 2011).

## Conflict of Interest Statement

The authors declare that the research was conducted in the absence of any commercial or financial relationships that could be construed as a potential conflict of interest.

## Abbreviations

AON, action observation network; ASD, autism spectrum disorder; CVE, collaborative virtual environment (aka shared virtual environment); DVE, desktop virtual environment; fMRI, functional magnetic resonance imaging; HFA, high-functioning autism; IVE, immersive virtual environment; SNN, social neural network; SVE, single-user virtual environment; TSI, transformed social interaction; VC, virtual characters; VE, virtual environment; VR, virtual reality.
